# Knowledge in motion: temporal dynamics of wild food plant use in the Polish-Lithuanian-Belarusian border region

**DOI:** 10.1186/s13002-024-00706-8

**Published:** 2024-07-12

**Authors:** Julia Prakofjewa, Matteo Sartori, Povilas Šarka, Raivo Kalle, Andrea Pieroni, Renata Sõukand

**Affiliations:** 1 Department of Environmental Sciences, Informatics and Statistics, Ca’ Foscari University of Venice, Venice, Italy; 2 Botanical Garden of Vilnius University, Vilnius, Lithuania; 3 University of Gastronomic Sciences, Bra, Pollenzo, Italy; 4 Estonian Literary Museum, Tartu, Estonia; 5 Department of Medical Analysis, Tishk International University, Erbil, Iraq

**Keywords:** Historical ethnobotany, Local ecological knowledge, Wild food plants, Diachronic comparison, Poland, Lithuania, Belarus, Knowledge circulation

## Abstract

**Background:**

Understanding how Local Ecological Knowledge (LEK) evolves over time is crucial for fostering social and environmental responsibility. This study aims to develop a conceptual model of plant knowledge circulation, providing insights into the temporal dynamics of LEK in the Polish-Lithuanian-Belarusian border region. It explores the key patterns and driving forces behind changes in the use of wild plants for food.

**Methods:**

Field research was conducted in 60 rural settlements across Podlasie Voivodeship (Poland), Vilnius Region (Lithuania), and Hrodna Region (Belarus). This included 200 semi-structured interviews and participant observation among two local communities, Lithuanians and Poles. To assess the temporal dynamics of wild food use, we performed a cross-ethnic, cross-border analysis over time, dividing the data into three major temporal dimensions: past, continuous, and recently acquired uses.

**Results:**

Of the 72 wild plant taxa reported by Poles or Lithuanians in the Polish-Lithuanian-Belarusian borderland, 47 were continuously used for food, 58 were utilised in the past, and 41 were recently acquired. Cross-country trends were similar, with Poland showing more past uses. Diachronic comparisons between Poles and Lithuanians in each studied country revealed no significant differences. Recently acquired taxa overlapped considerably with those used continuously and in the past. The most diversely utilised taxa showed the greatest overlaps. By observing the movement of specific plant taxa within various time dimensions, we distinguished overlapping flow variations: retention (3 taxa), decay (11), invention (8), stagnation (17), revitalisation (6), re-invention (3), and knowledge in motion (24). Shifts in the use of wild food plants were influenced by changes in environmental conditions, governmental policies, cultural practices, and economic factors.

**Conclusion:**

The findings of this study have important implications for improving methods of tracking changes in LEK and enhancing our understanding of the relationship between people and nature. Our results underscore the importance of considering knowledge circulation over time in different directions. Recognising the various stages of knowledge circulation might help in pursuing sustainable solutions that balance the needs of human communities with environmental protection.

## Background

Studying nature-related knowledge is essential for developing a deeper and more detailed understanding of the world around us. This knowledge is not a static compilation of established assertions [1], but a multifaceted and interrelated set of ideas, concepts, and theories that evolve and undergo transformations over time [2]. Ecological knowledge is deeply entrenched in social, cultural, and historical contexts, shaping how local communities create, communicate, and appreciate the surrounding environment.

LEK (local ecological knowledge) is a knowledge system formed through interactions between people and the environment over time and space. It is characterised by its flexibility and adaptability in the face of changing social and environmental conditions [3, 4] and is in a perpetual state of motion, continuously evolving to adapt to new circumstances.

One current trend in ethnobotanical studies involves exploring the dynamics between human populations and plant-based foods and medicines with historical significance in maintaining human nutrition and health [5]. There is growing interest in studying traditional ecological knowledge (TEK) as a part of LEK and the sustainable use of wild plants worldwide [6–8]. This research helps preserve and revitalise this knowledge for future generations [9, 10]. By examining traditional cultural practices related to wild plant use, we can identify effective and ecologically responsible approaches used by local communities for centuries [11]. Previous studies have shown that as modern societies become more industrialised and urbanised, TEK is often devalued or lost because people adopt more contemporary practices and technologies [12, 13]. Scholars have also highlighted a strong inverse correlation between ecological knowledge and income level [14, 15]. Additionally, several investigations have explored the potential effect of formal education on TEK loss [16, 17].

More generally, LEK transmission in highly literate communities may involve different processes, sources, and factors compared to communities with lower literacy levels. In post-Soviet territories, a considerable proportion of LEK is now transmitted through written and visual sources [18]. Books have played a valuable role in the transmission and preservation of LEK [19]. Highly literate communities often have greater access to technology, which can be used to construct and transmit new LEK [20]. Digital media and online platforms are now widely used to share information and experiences about traditional knowledge and new practices [21–23]. Conversely, the migration of younger generations from rural areas to urban centres has disrupted the oral transmission of TEK, leading to its gradual loss [24].

Most research on the utilisation of wild plants has documented that knowledge evolves over time as new discoveries are made [25], other practices are abandoned, technological advancements are developed [26], and cultural shifts occur [27, 28]. Several ethnobotanical studies have generally compared remembered/past and current/present uses of plants [29–35]. However, previous research has not yet addressed the documentation of changes in LEK in terms of the potential continuity and discontinuity between past and present uses of plants. Depending on how LEK is managed and experienced by local communities, there might be a loss of knowledge, the spread of new uses, and the flow/permanence of past plant knowledge.

The Polish-Lithuanian-Belarusian borderland constitutes an excellent case study of temporal changes in the use of wild food plants. This area has been a site of frequent geopolitical struggles, including invasions, wars, and border disputes. It represents a historically united, multicultural, and multi-linguistic region. The use of wild plants for food in this study area has been analysed primarily from a historical perspective, exploring archival written sources published at the end of the nineteenth century and the beginning of the twentieth century [36–38]. Despite several studies focusing on the use of wild plants in the tri-border area, the food domain was understudied in this period [39, 40] and, therefore, poorly understood. Several contemporary studies have been published on specific sites of the border triangle, highlighting the impact of socio-economic and cultural changes on the current perception of gathering activities and wild food resources [41–43].

In this paper, we contribute to the body of previous research [44] by exploring the temporal dynamics of wild food plant use. Drawing on data from the Polish-Lithuanian-Belarusian border area, we test the hypothesis that recognising the various flow variations in knowledge circulation provides a more precise investigation of the dynamics associated with using wild plants over time. To achieve this, we have established the following specific objectives: (1) to assess changes in local wild food plant knowledge within the border area of Poland, Lithuania, and Belarus, (2) to identify the key factors that may have shaped the documented knowledge; and (3) to propose a conceptual model concerning plant knowledge circulation over time. By enhancing comprehension and interpretation of the shifts in the relationships between people, knowledge, and nature over time, we seek to facilitate more effective endeavours in fostering biocultural diversity in fragile border areas.

## Materials and methods

### Study area and historical background

The Polish-Lithuanian-Belarusian borderland is located at the crossroads of Central and Eastern Europe. Historically, this border region has been a contested area and remains a geopolitical hotspot. The landscape is dominated by a hilly lake district shaped by Baltic glaciation [45] and includes various types of landscape, including forests, lakes, rivers, and wetlands [46]. The region's distinctiveness lies in its transitional vegetation, which is relatively homogenous but features a combination of Central European and Northeast European species, particularly those of a boreal nature [47].

**Fig. 1:**
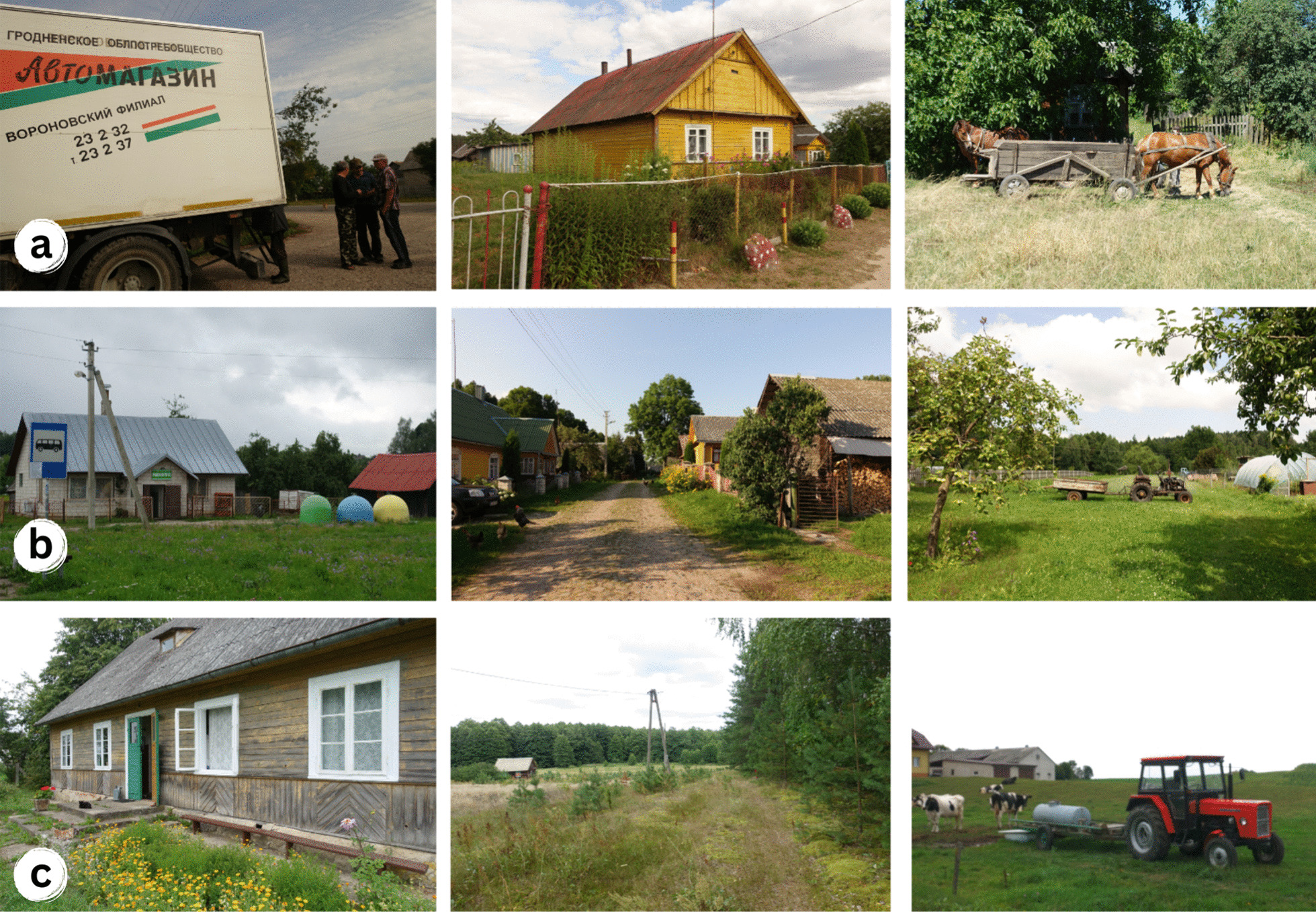
Field site conditions: **a** Belarus, **b** Lithuania, **c** Poland. Credit: Julia Prakofjewa, 2018–2019

While the border region is predominantly rural, it also includes several industrial centres, such as Suwałki, Hrodna, and Vilnius, which have a history of manufacturing, including textiles, food processing, and machinery production. Economic development in the region has been uneven, with some areas experiencing rapid growth and others lagging. There are high unemployment rates, particularly among young people, and significant wealth and income disparities exist between urban and rural areas. Despite challenges such as political instability, economic inequality, and environmental degradation, the region continues to be a vital and dynamic component of the European landscape [48] (Fig. 1).

The study region has a complex history of political, economic, and cultural interactions among Poles, Lithuanians, Belarusians, Jews, Tatars, Ukrainians, and other ethnic groups. This diversity has contributed to a rich cultural heritage but has also led to tensions and conflicts, particularly during political upheavals [49–51].

From the 14th to the middle of the twentieth century, Poland, Lithuania, and Belarus shared significant historical events. The region was at the crossroads of various empires and state units, including the Polish-Lithuanian Commonwealth, the Russian Empire, and the Soviet Union [52]. Before World War II, Western Belarus was a part of Poland, but in 1939, following the Soviet invasion of Poland, it was incorporated into the USSR as a part of the Byelorussian Soviet Socialist Republic. Similarly, in 1940, the Soviet Union annexed Lithuania and integrated it as the Lithuanian Soviet Socialist Republic. Both Lithuania and Belarus remained part of the Soviet Union until they declared their independence in 1990 and 1991, respectively, after the collapse of the USSR [49]. During the Cold War (1947–1991), Poland was under Soviet influence as a satellite state and a member of the Warsaw Pact. Poland experienced Soviet control and intervention, leading to the suppression of political opposition, media censorship, and the control of security services. The emergence of the Solidarity trade union movement in the late 1970s challenged the communist government, resulting in political and social upheaval and the establishment of a democratic government in 1989 [53]. Currently, Poland, Lithuania, and Belarus are independent states, each with its own governments and political systems.

Intense social, political, and economic transformations during the twentieth century have reshaped the structure of land use in the region. These changes have led to a reduction in agricultural land through the abandonment of unproductive land, resulting in spontaneous afforestation in some areas and deforestation in others. There has also been a decline in arable land, leading to a decrease in crop acreage and an increase in orchards, meadows, and pastures [54]. The functioning of wetlands, especially peatlands, in former Soviet territories has been significantly impaired, resulting in disrupted water flow regimes, altered carbon and nutrient cycles, changes in vegetation cover, and the loss of biodiversity [55].

### Data collection

**Fig. 2:**
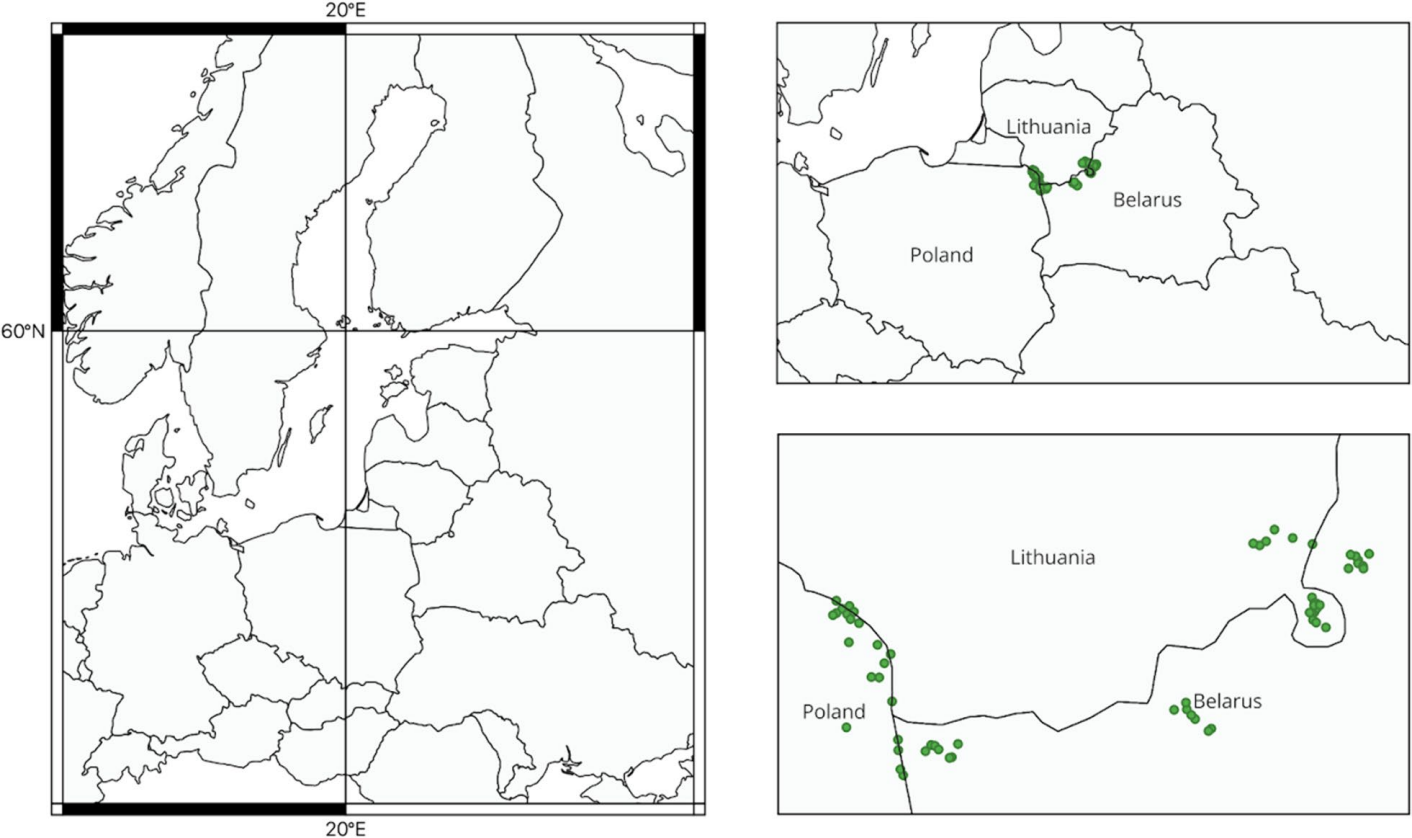
Map of the study area: border region between Poland, Lithuania, and Belarus. Designed with QGIS 3.22 'Białowieża'

The ethnobotanical study was undertaken between June and August 2018 and July and September 2019. The research took place in 60 rural settlements across Hrodna Region (NW Belarus), Vilnius Region (SE Lithuania), and Podlaskie Voivodeship (NE Poland) (Fig. 2). We focused on intensive fieldwork within two local communities: Polish and Lithuanian. In each case study, we primarily chose rural settlements near forests. In the tri-border area, the Lithuanian population is smaller and more compactly settled, leading us to conduct research in villages that are quite remote from Polish settlements.

In total, we carried out 200 semi-structured interviews, each lasting between 30 min and 3 h, focusing on the use of wild plants for food, medicine, and other purposes. Wild food plants are defined as those growing without deliberate cultivation (see [56]). The interviews occurred in various settings, including homes, gardens, and nearby forests. Additionally, participant observation was performed whenever possible during the collection, preparation, and cooking of wild plants.

Half of the interviewees in Belarus and Lithuania were visited twice to ensure comprehensive data collection. To promote the most favourable settings during conversations, all participants were interviewed in their preferred language. The first and third authors' proficiency in the local languages obviated the need for translators.

The interviewees were conveniently selected, with the primary aim of engaging individuals of Polish and Lithuanian origin to share their personal experiences and reflections on the use of wild plants. A minimum age of 40 was chosen so that interviewees could provide insight into plant use changes in the latter half of the twentieth century. We interviewed 95 Lithuanians and 105 Poles, with average ages of 68.54 and 72.07 years, respectively. Most of these individuals are now retired. The majority of interviewees in Poland were formerly small-scale farmers, while those in Belarus and Lithuania had worked on *kolkhozes* (collective farms), had subsistence gardens, and engaged in husbandry. About 25% of interviewees were employed as teachers, nurses, and administrative workers.

Oral prior informed consent was obtained before the interviews, and about 75% of the interviewees from Poland and Lithuania provided written informed consent. The ethical guidelines prescribed by the International Society of Ethnobiology (ISE, 2006) [57] were rigorously followed. Furthermore, we acquired ethics approval from the Ca' Foscari University of Venice Ethics Committee. Transcripts of all interviews were created, preserving linguistic and metacommunicative subtleties to enhance transparency and ensure the quantitative and qualitative analysis can be reproduced.

Whenever possible, we collected wild herbaceous voucher specimens with the help of our interviewees. Plant names are provided according to the Plants of the World Online database [58] and Flora Europaea [59]. Plant families correspond to Stevens [60] (2001 and onwards). The voucher specimens were deposited at Ca' Foscari University of Venice (Italy). Specimens and dried plant samples collected from Lithuania bear accession codes DZULT01–DZULT126 and DDZULT01–DDZULT45, and specimens and dried plant samples collected from Poland bear accession codes DZUPL001–DZUPL107 and DDZUPL01–DDZUPL39.

### Data analysis

Transcripts and notes collected from interviews were manually organised and coded in Microsoft Excel in the form of Detailed Use Reports (DUR) (see [31]).

For diachronic comparison, the data was divided into three temporal dimensions:

The "past" includes all uses noted by the interviewees as being from the post-war period to the collapse of the Soviet Union and the fall of the Communist regime in Poland, as well as memories of practices that were more recently abandoned in the 1990s and early 21st century.

The "continuous" category refers to the uninterrupted use of wild plant taxa for food from childhood to the present time.

The "recently acquired" category includes plant uses that have been recently adopted or those for which the use method has changed (e.g., frozen birch sap).

The proportion of uses in the different temporal dimensions was calculated for every taxon. Quantitative analysis and graph plotting were performed with Microsoft Excel and R 4.2.2 software using various CRAN packages [61]. To illustrate temporal changes within diverse communities, we used RAWgraph (rawgraphs.io) and euler*APE* software [62].

### Quantitative results

**Fig. 3:**
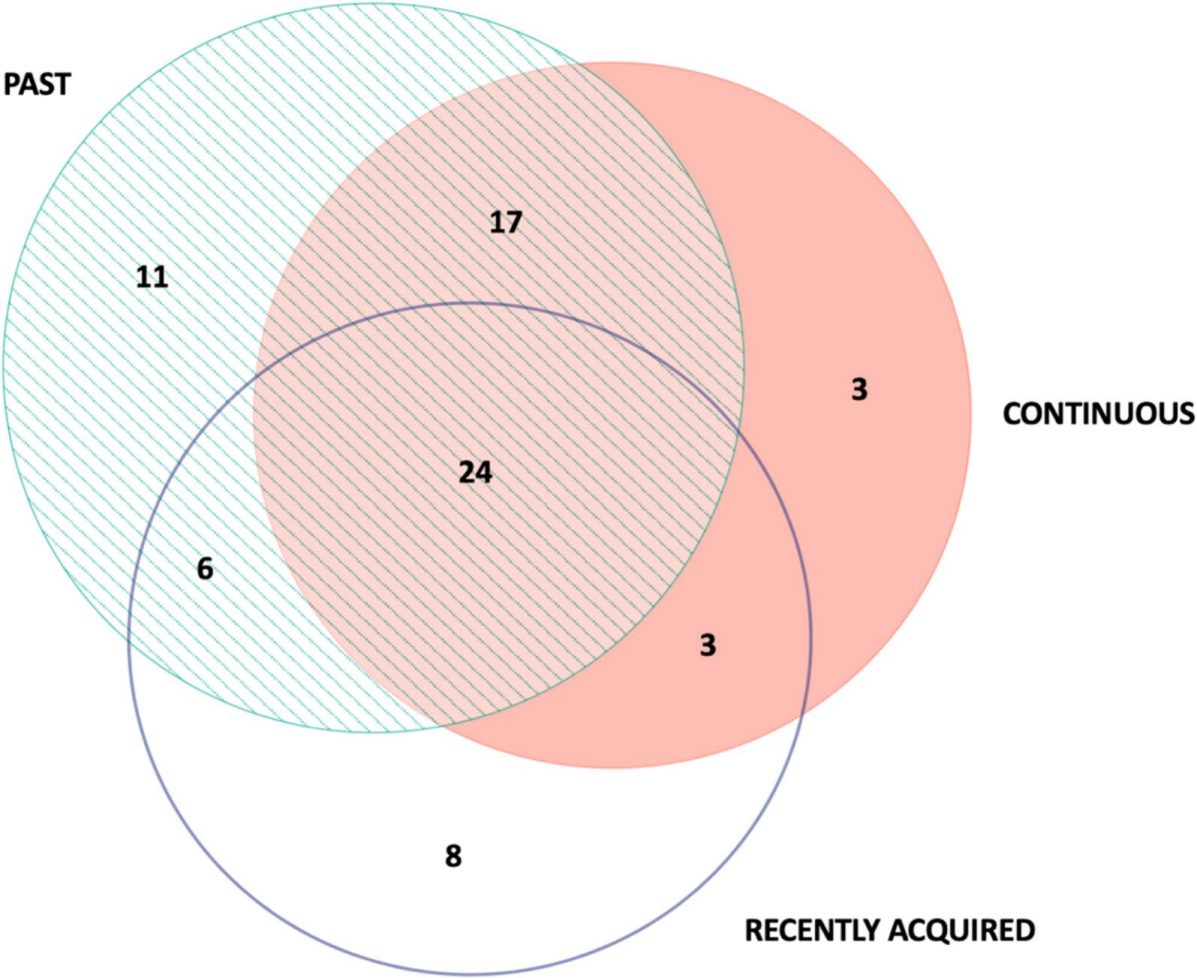
Venn diagram comparing the past, continuous, and recently acquired plant taxa as reported by the interviewees

Of the 72 wild plant taxa reported by either Poles or Lithuanians as used for food in the Polish-Lithuanian-Belarusian borderland, 47 have been continuously in use by at least one person. Fifty-eight taxa were named as used in the past by at least one person, while 41 taxa were reported as recently acquired by at least one person (Fig. 3). Each interviewee mentioned an average of approximately 9.625 wild plant taxa, with the number of species known ranging from a minimum of 1 to a maximum of 25.

**Fig. 4:**
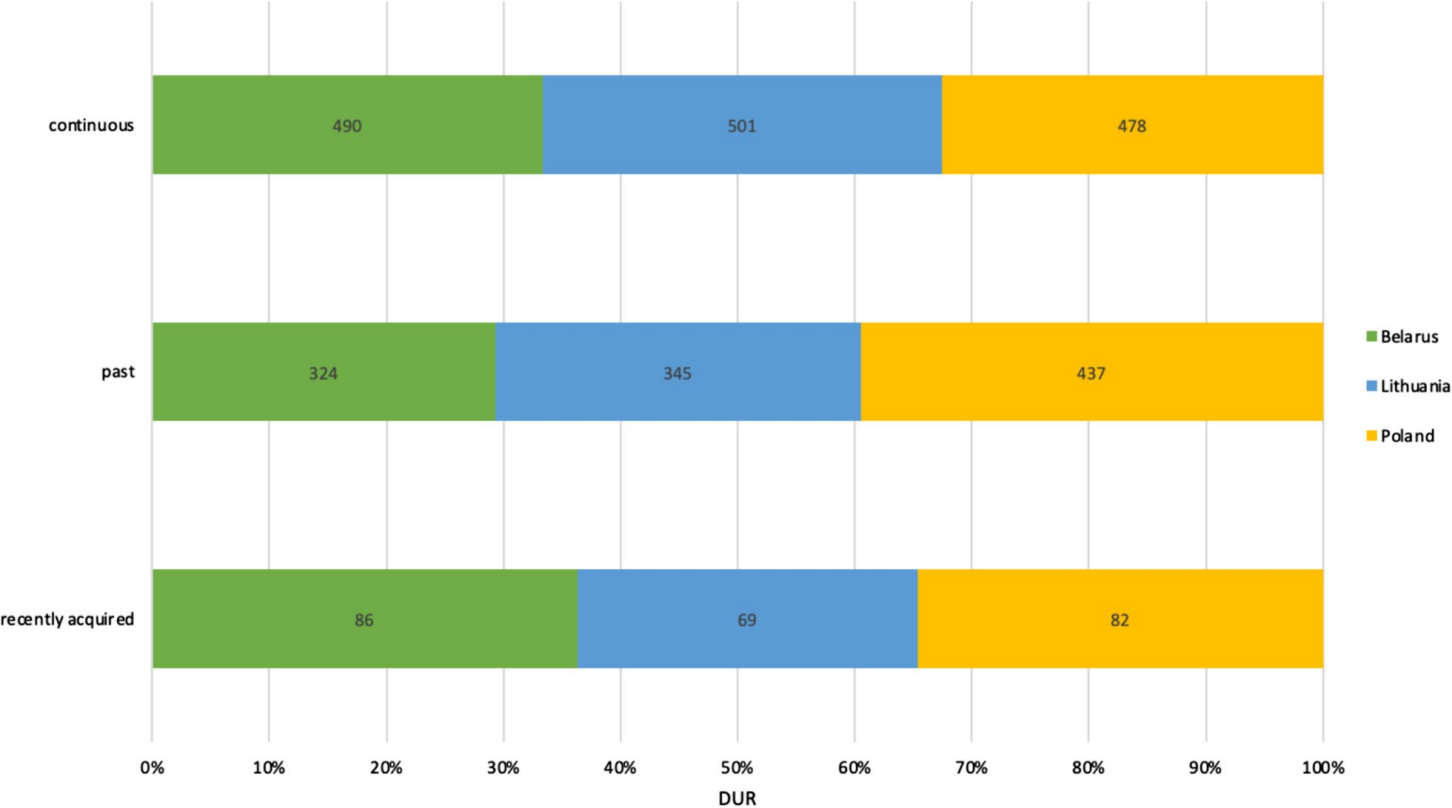
Comparison of DUR by country

Fifty-two percent of all DUR refer to plant uses practised throughout the lifetime of the interviewees, while 39% of DUR refer to uses that remained in the past and 8% of DUR have been acquired recently. The total number of continuous DUR is proportionally higher in Belarus and Lithuania compared to past DUR in these two countries. In Poland, past DUR were proportionally slightly more numerous than continuous DUR. The cross-country trends are similar, except that Poland has a proportionally higher number of past uses (Fig. 4).

**Fig. 5:**
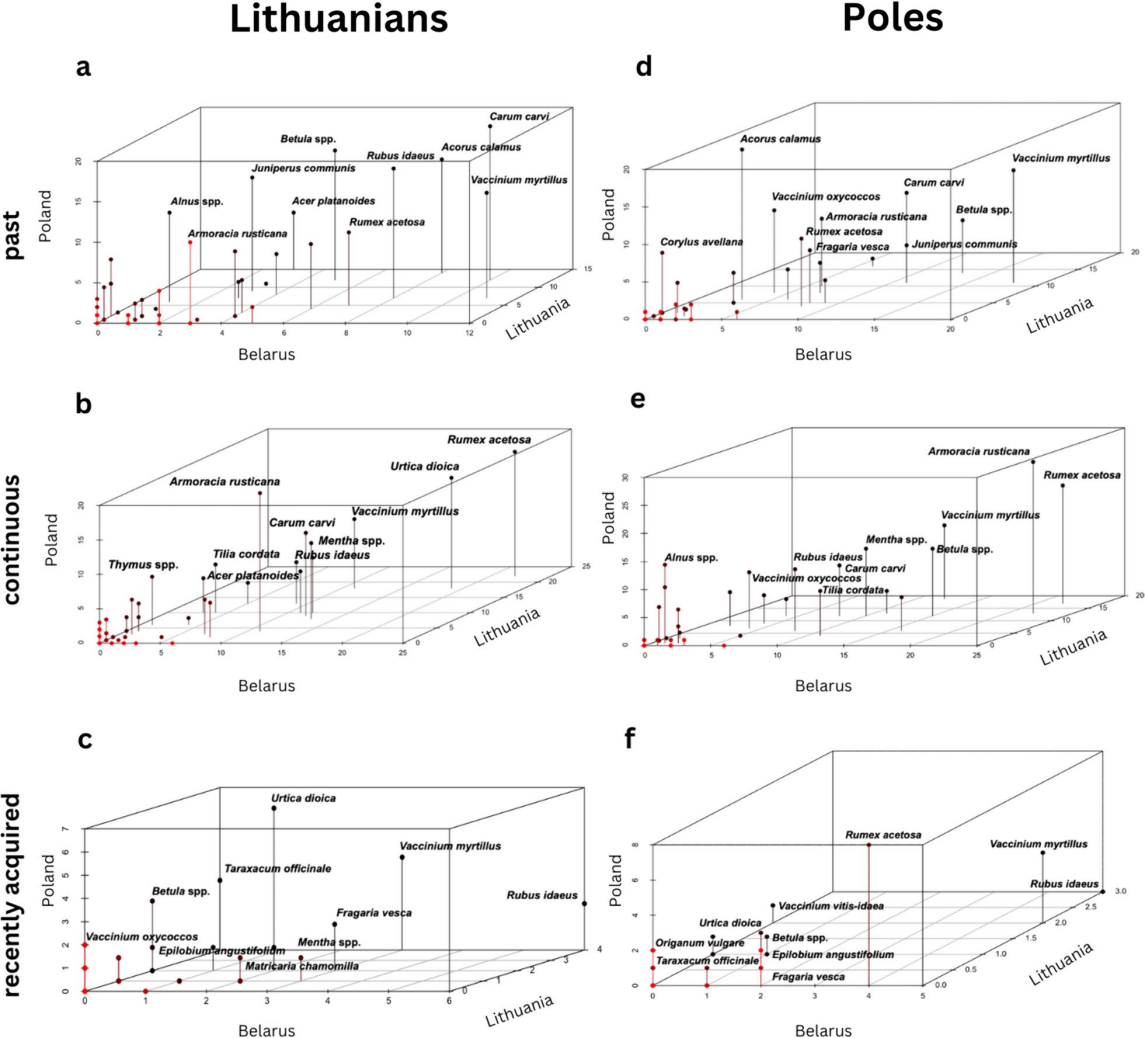
Scatter plots of food plant taxa recorded in Poland, Lithuania, and Belarus among Lithuanian and Polish communities; designed in RStudio.

The diachronic comparison between Poles and Lithuanians in every studied country revealed no remarkable differences (Fig. 5). The comparison of recently acquired plant taxa and those used continuously and only in the past showed considerable overlap for both the cross-cultural and cross-country comparisons. The taxa with the greatest use overlaps were generally those that were most diversely utilised.

Specifically, Lithuanians and Poles in all case studies showed considerable overlap, with 13 species used in the past and 17 continuously used. The most heterogeneous local knowledge regarding wild food plants was observed between Poles from Poland and Lithuanians from Lithuania for both past and continuous uses. Knowledge was most homogenous between Poles from Belarus and Poles from Poland for past uses and between Poles living in Belarus and Lithuanians from Lithuania for continuous uses. For the uses that remained in the past, the two ethnic groups of Lithuanians and Poles shared cross-border 16 and 17 taxa, respectively, and most of the top five taxa overlapped, except for *Acorus calamus* for Lithuanians (Fig. 5a) and *Juniperus communis* for Poles (Fig. 5d).

Poles and Lithuanians shared 19 plant taxa for life-long uses across the three countries. The most homogeneous level of life-long knowledge was between Lithuanians from Lithuania and Lithuanians from Belarus (Fig. 5b) and between Poles from Belarus and Poles from Poland (Fig. 5e). The top five taxa used throughout life differed in *Rubus idaeus* and *Urtica dioica* among Lithuanians and *Armoracia rusticana* and *Mentha* spp. among Poles.

Lithuanians had recently learned to use eight common plant species (Fig. 5c), whereas Poles shared only two taxa (Fig. 5f). The majority of new uses were related to changes in preservation technology. For instance, the introduction of modern freezing techniques in the 1970s allowed for the preservation of wild berries, which were previously only used fresh or dried, thereby expanding their culinary applications throughout the year.

**Fig. 6:**
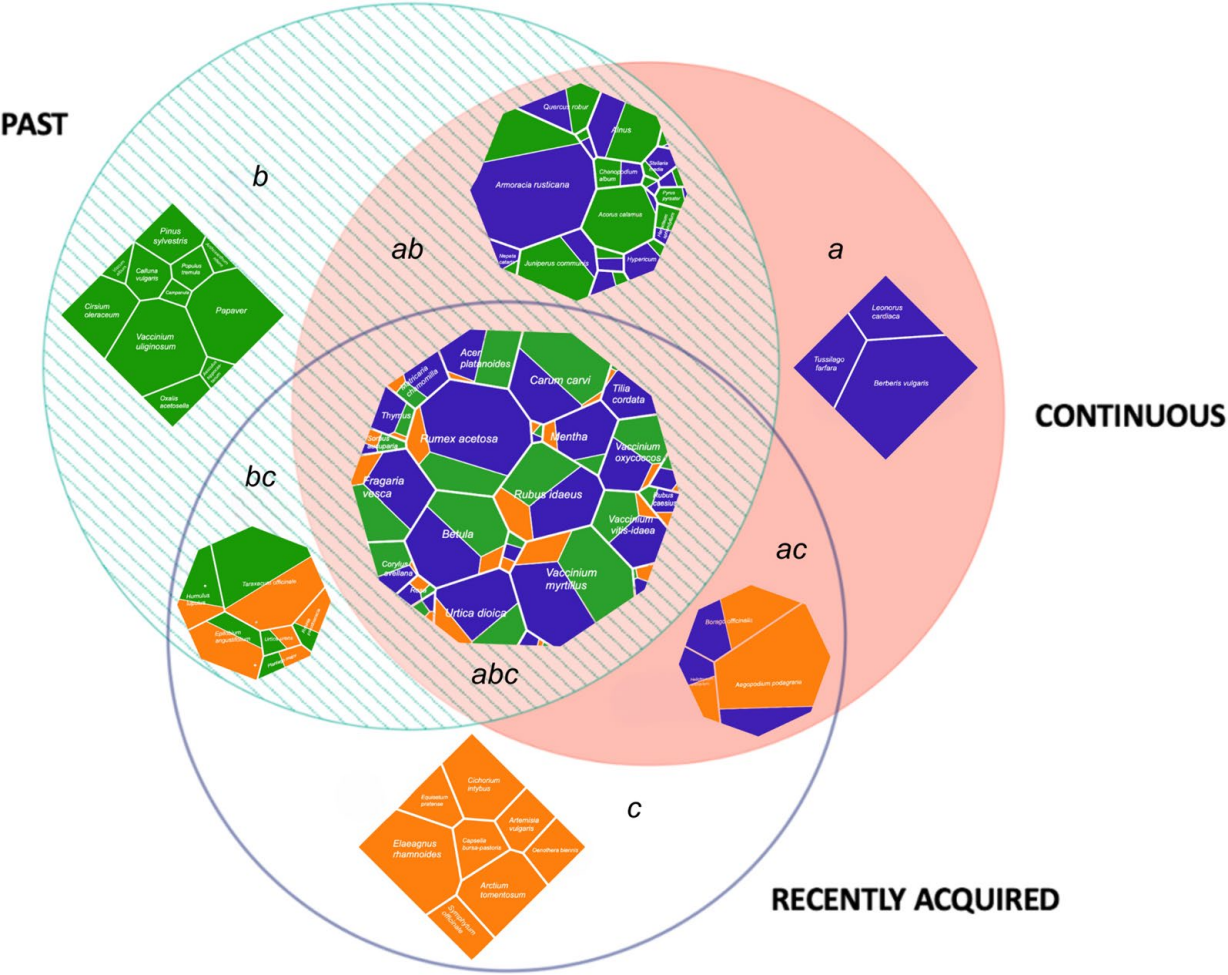
Temporal dynamics of wild food plant use in the Polish-Lithuanian-Belarusian borderland: **a**—retention; **b**—decay; **c**—invention; **ab**—stagnation; **bc**—revitalisation; **ac**—re-invention; **abc**—knowledge in motion. Green indicates the proportion of past, orange—recently acquired, and blue—continuous uses of wild plants for food

We distinguished overlapping circulation areas by observing the movement of specific plant taxa within past, continuous, and recently acquired time dimensions. Consequently, we recognised specific flow variations: retention, decay, invention, stagnation, revitalisation, re-invention, and knowledge in motion (Fig. 6).

The majority of documented DUR in the Polish-Lithuanian-Belarusian borderland belonged to plants reported in all three temporal dimensions (Fig. 6abc). The predominant taxa in the domain include *Rumex acetosa*, *Vaccinium myrtillus*, *Betula* spp., *Vaccinium vitis-idaea*, *Fragaria vesca*, *and Urtica dioica*, confirming the high cultural significance of these species for the studied ethnic groups (see [44]). The taxa in this domain were mostly reported as continuously used, with a small proportion of recently introduced uses.

The second most mentioned flow variation represented plants that some interviewees have continuously used while others have already abandoned them (Fig. 6ab) with no recently acquired uses. Here, some taxa have been in continuous use for the majority of the interviewees (e.g., *Armoracia rusticana*, *Hypericum* spp., *Betula* spp*.*, *Nepeta cataria*, *Stellaria media*)*,* while others (like *Juniperus communis*, *Acorus calamus*, *Pyrus pyraster*) are mainly only remembered by most of the interviewees who named them.

The remaining 4.6% of DUR are divided between five minor temporal flows. Specifically, of the 11 taxa mentioned as utilised only in the past context (Fig. 6b), the most intensively used were *Vaccinium uliginosum*, *Papaver* spp*.*, *Cirsium oleraceum*, *Pinus sylvestris*, *Calluna vulgaris*, and *Oxalis acetosella.* Among the newly introduced species (Fig. 6c), we can highlight *Elaeagnus rhamnoides*, *Cichorium intybus*, and *Arctium tomentosum*. The taxa combining past and newly introduced uses (Fig. 6bc) included *Taraxacum officinale*, *Epilobium angustifolium*, and *Humulus lupulus*, for which new uses predominated only for *Epilobium angustifolium*. In contrast, in the flow variation where continuous and recently acquired uses are combined (Fig. 6ac), new uses predominated for three plants: *Helichrysum arenarium*, *Borago officinalis*, and mainly *Aegopodium podagraria*. *Tussilago farfara*, *Leonorus cardiaca*, and *Berberis vulgaris* were mentioned by our interviewees as continuously used (Fig. 6a).

## Qualitative results and discussion

### Knowledge circulation in the Polish-Lithuanian-Belarusian borderland

We detected significant changes in the knowledge of wild food plants in the border region shared by Poland, Lithuania, and Belarus. We observed that most taxa demonstrated a movement of knowledge that includes past, ongoing, and newly learned practices. This can be defined as *knowledge in motion*, indicating a continuously evolving flow variation. The mentioned plants were of considerable importance in all three countries that were studied. This importance was assessed using the cultural importance index, which quantifies the significance of these plants based on their usage and the continuity of their knowledge (see [44]). Among the most common food taxa recorded were *Urtica dioica* and *Rumex acetosa*. Their use in soups was also supported by historical written sources, showing a longstanding tradition in the region (see [36–38]: *“Viasnoj to, nu što dla supa viasnoj—eta pieršym dziełam, maładaja krapiva. A bolš što jašče. Ščaviel. Ale ščaviel eta paźniej uže. Pieršym dziełam—krapiva. Eta abizacielna. Ščaviel jašče i na zimu stavim”* [*In spring, well, for soup in spring, the first thing is young nettles. And what else? Sorrel, but sorrel is used later. First of all, nettle. It is a must. We also conserved sorrel for winter*] (Belarus, Polish man, 60 years old).

**Fig. 7:**
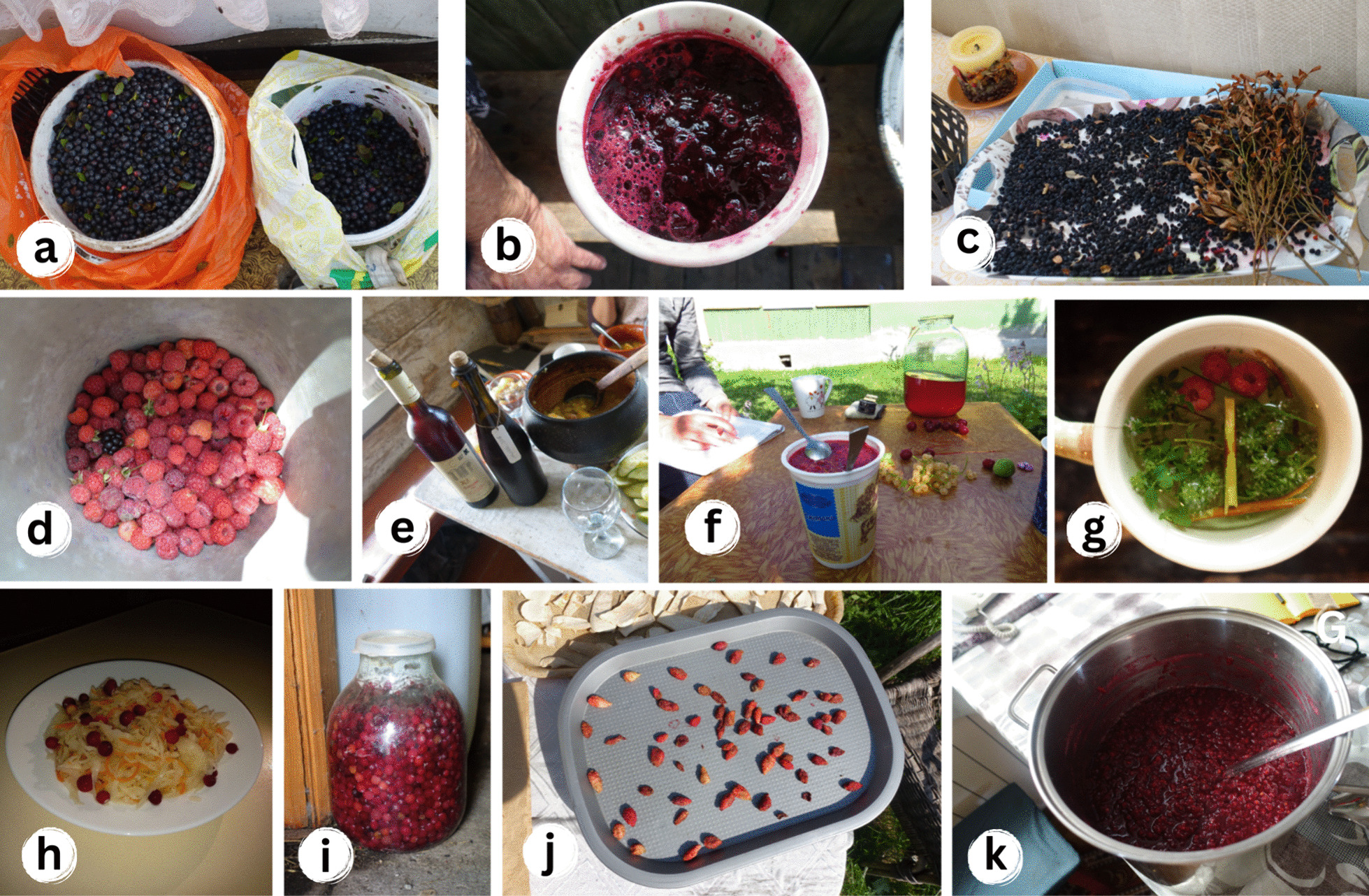
The use of wild fruits in motion: **a** Just gathered *Vaccinium myrtillus*, Lithuania; **b** Jam from *Vaccinium myrtillus*, Belarus; **c** Drying of *Vaccinium myrtillus*, Lithuania; **d** Collecting of *Rubus idaeus*, Belarus; **e** Wine from *Rubus idaeus*, Belarus; **f** Frozen jam and compote from *Rubus idaeus,* Belarus; **g** Recreational tea with *Rubus idaeus*, Lithuania; **h** Sauerkraut (fermented cabbage) with *Vaccinium oxycoccos*, Belarus; **i** *Vaccinium oxycoccos* soaked in water for winter, Lithuania; **j** Drying of *Fragaria vesca*, Lithuania; **k** Making jam from *Vaccinium vitis-idaea,* Poland. Credit: Julia Prakofjewa, 2018–2019

We found that the frequently cited wild berries, such as *Vaccinium myrtillus, Rubus idaeus, Fragaria vesca, Vaccinium oxycoccos*, and *Vaccinium vitis-idaea,* have circulated within the three time dimensions with varying intensities. Over time, methods of use and preparation techniques have undergone changes, while the enduring value of these plants remains remarkably high in terms of their utility and continued use (Fig. 7).

Interestingly, we recorded the essential role of *Vaccinium myrtillus* during food shortages in Poland during WWII: "*[W 1939] … uciekaliśmy, bo tutaj wisiał taki samolot, no samolot wisiał. W powietrzu. Ale słuchamy, krzyczy ktoś, żeby nie iść w tą stroną. Tylko iść, bo tu jeszcze nie ma tych Niemców. A iść w tą stroną. Bo to już Ruskie… To my biegiem i przez ten gościniec. I poszli do tego lasu. Przyszli do tego lasu, Jezus Maria, a nic nie mamy ze sobą. Ale gadali, gadali te ruskie, tylko tego już oni idą, już oni idą i wszystko… A my z sobą tylko krowy mieli… I gotowali. A co? Gotowali kartofla. A ta kartofla, to była taka była… taka malutka była, taka malutka była. No i tu, ta kartofla i z jagodami gotowali. Z czarnymi. Bo tutaj pełno czarnych…* Jestem wciąż wdzięczna tym jagodom za to, że nas uratowały" [*[In 1939] … we were running away because there was a plane like this hanging here, a plane was hanging here. In the air. But we were listening, someone was shouting not to go down this road. But to use another one, because those Germans weren't here yet. And go down this road. Because it was already the Russians… We ran along this road. And we went into this forest. We got into the woods, Jesus Maria, and we had nothing with us… And we only had cows… But they were talking, they were talking, these Russians, only that they were going, they were going and everything. And we cooked. And what? We cooked potatoes. And these potatoes, they were so … so tiny, so tiny. And here, these potatoes were cooked with blueberries. With blueberries. Because there were a lot of blueberries… I'm still grateful to those blueberries. For saving us all…*] (Poland, Polish woman, 92 years old). The same use during WWII was confirmed by other senior interviewees from Poland.

From an ecological standpoint, specific berry species growing in the forests, such as *Vaccinium myrtillus*, *Vaccinium vitis-idaea*, *Rubus idaeus*, and *Fragaria vesca*, along with synanthropic plants (including certain weeds) like *Urtica dioica* and *Rumex acetosa*, demonstrate greater resilience and suitability for use as food plants. Conversely, among non-berry species, there are several whose use has been abandoned over time, such as *Pinus sylvestris*, *Calluna vulgaris*, and *Oxalis acetosella*.

Sharing information about the properties and uses of plants between different ethnic groups often results in heightened awareness, improved utilisation, and more sustainable practices among all local communities. For instance, the flowers of *Sambucus nigra* were historically fried in batter by Poles (see [37, 63]). During fieldwork, we also documented the same use among Lithuanians in Poland: "*Petruk, kap tas vadinasi, juodi tie mūs bezai? Nu aš sakau, aš pavadinimų nežinau. Tokios kekės žydi žiedų, balti… Šeivamedis. Iš laukų. [renka] Jų namie nesodinam niekas… O žiedus tai kepėm. Nu jis kaip nusiima aplinkui toks gražus, kaip grybą pavyzdžiui, tą in kokį prieskonį, in kiaušinį in džiovytus tuos džiūvėsiukus ir an keptuvės. Jis va šitokiu būdu visai gerai kepasi.*” [*Petruk, how is it called [in Lithuanian], our black bezai? Well, I’m telling you, I do not know the names. Such clusters of white flowers bloom together… Šeivamedis. [They collect] from fields. None of us plants them at home… And flowers we baked. Well, when you pluck it [flower], it is so round and nice, like a mushroom; for example, you put it in some seasonings, into an egg, in breadcrumbs, and into a frying pan. In this way, it cooks pretty well*] (Poland, Lithuanian woman, 70 years old). According to our Lithuanian interviewees from Poland, they learned about this particular plant use from a promotion they saw on Polish national TV. As a result, they had difficulty recalling the local name and primarily used the common Polish name. Nowadays, the flowers/fruits of *Sambucus nigra* are mainly used for making recreational tea or non-alcoholic drinks throughout the border area.

Svanberg et al. [64] suggested that tree sap extraction was predominantly historically practised in regions with abundant *Betula* spp. and *Acer platanoides* populations, as these species tend to yield significant quantities of sap. Modern ethnobotanical studies have shown that fresh and processed drinks made from tree sap have had a relatively high informant consensus factor in Belarus, both historically and today [65]. Birch sap has been widely used fresh and fermented in Lithuania for centuries [64]. In the eastern part of Poland, the use of birch trees for sap was widespread in the nineteenth century (see [37]), but by the mid-twentieth century, it was (nearly) obsolete and practised mainly by boys as a form of spring entertainment [36, 64, 66]. The use of *Acer platanoides* was popular in Belarus and Lithuania but documented as very rare in Poland, mainly occurring in the eastern part. of the country. It did gain popularity after 1945 in areas of north-eastern Poland, where people from present-day Lithuania and Belarus were resettled [64].

Our field research has confirmed the changes recorded in previously published sources. Specifically, in Belarus, we documented a variety of continuous practices of sap preservation (fermentation and pasteurisation), along with its active fresh use. Belarusian interviewees were actively experimenting with adding different ingredients to birch sap in order to change its colour and flavour. For instance, along with the use of *Mentha* spp. and *Ribes nigrum* leaves, people were adding slices of different citrus fruits, raisins, citric acid, and even caramel.

In Lithuania, both studied communities were still actively gathering and processing sap. We recorded an equal temporal distribution of DUR between present and past uses. The majority of interviewees confirmed the historical use, namely the fermentation of birch sap with the addition of *Avena sativa* [67]. They described the whole process very precisely, which, though not practised, still remains in the collective memory: "…*su avižom ten būdavo beržų tą sulą kai suleidžia, tai užpila, avižas dėdavo, jos tokią plutą sudaro, sudygsta, kaip atžalos tokios gaunasi avižų ir ta pluta uždengia visą sulą ir jinai rūgsta, ir jinai labai skani*" […*with oats, they used to make birch sap. After collecting the sap, they used to put oats on top. They made such a crust. Then the oats sprout. Then, the shoots of oats were there as a result. And that crust covered all the sap, and it fermented. It is delicious*] (Lithuania, Lithuanian woman, 56 years old). The majority of interviewees from Poland reported the occasional and continuous use of *Betula* spp. for sap. They mentioned using it mainly as a fresh beverage, which was frozen for the winter but not fermented. Overall, we observed a gradual decline in the use of fermentation techniques in all three countries, albeit to varying degrees. Interviewees from Belarus reported finding the sap available in stores much tastier than the fermented one. Most interviewees stated that they no longer ferment birch sap because they dislike the taste.

**Fig. 8:**
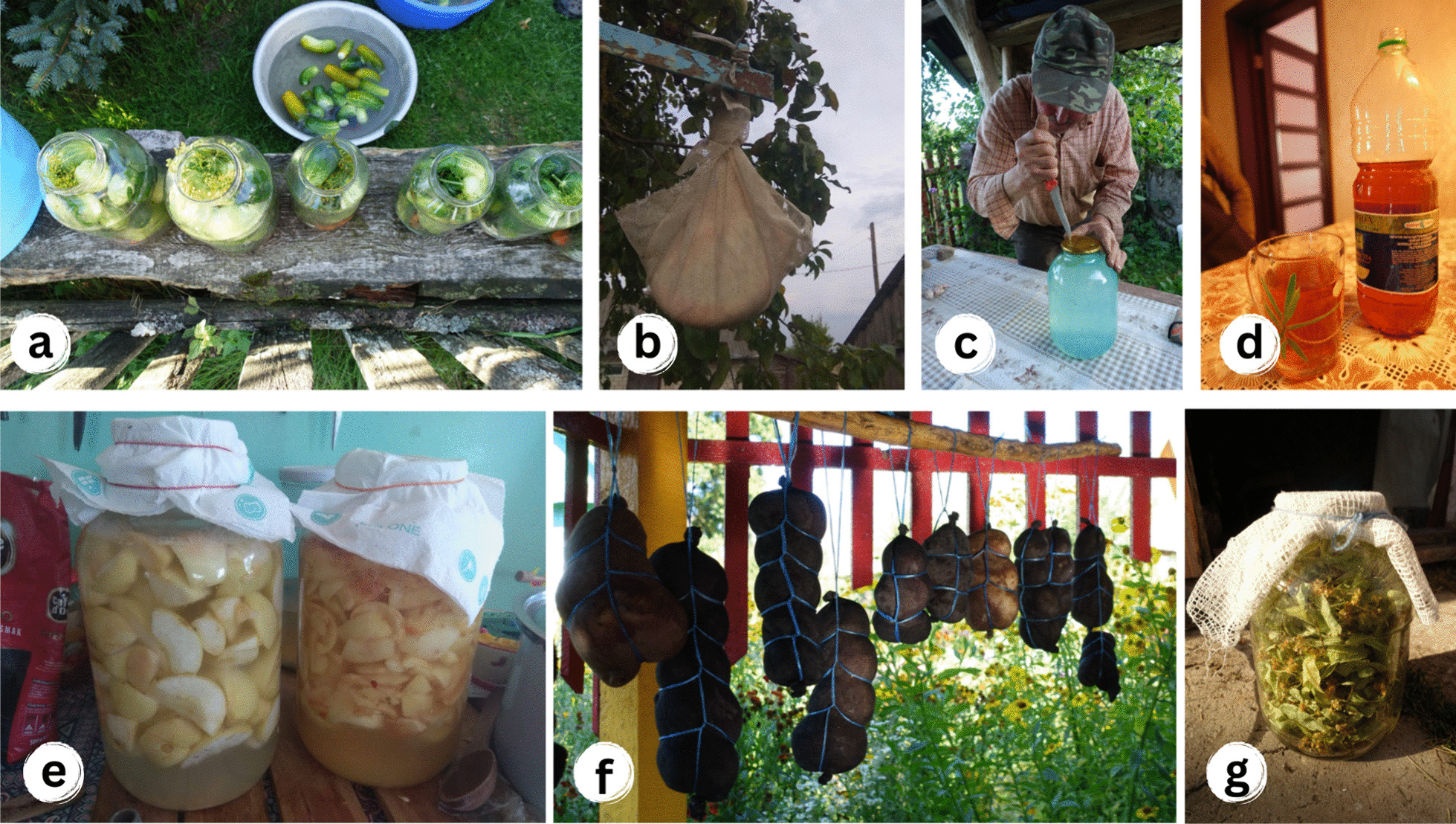
Wild food plants that are traditionally consumed in the study area: **a** Preparation of pickled cucumbers with roots of *Armoracia rusticana*, Lithuania; **b** Preparation of home-made cheese with *Carum carvi*, Belarus; **c** Birch sap, Lithuania; **d** Sap from *Acer platanoides*, Belarus; **e** Vinegar from wild apples, Poland; **f** Dried sausages with *Origanum vulgare*, Belarus; **g** Dried *Tilia cordata* prepared for recreational tea, Belarus. Credit: Julia Prakofjewa, 2018–2019

Our case studies have revealed a specific flow variation that overlaps between past and continuous uses, which could be recognised as a *stagnation of knowledge*. While some uses of plants in this flow were predominant in the past and continue to be integral to ongoing traditions (Fig. 8), others have lost their significance for the studied communities, remaining only in the historical written records. In such cases, the plants are still used, but some of their traditional uses have disappeared. For instance, in responses to Polish botanist Józef Rostafiński's (1883) questionnaire, the use of *Armoracia rusticana* was documented for the preparation of "*salad with vinegar*" (see [37]). The lexicon of Slavic beliefs and customs written by the Polish ethnographer Adam Fischer in the 1930s reported that this plant was used only in the medicinal domain (see [38]). Nevertheless, our fieldwork results have shown the great cultural importance of *Armoracia rusticana* among Polish and Lithuanian communities and its predominant uninterrupted use as food. Roots and leaves were used as a popular seasoning for lactofermented cucumbers and meat. More than half of the interviewees emphasised that they still make horseradish relish as a special ritual dish for Easter. The recorded stagnation in the tradition of using this plant can be attributed to several factors, including its gradual disappearance from the cultivated landscape in all three countries [66, 68], as well as the effort involved in making seasoning and the availability of ready-made options in stores: "*Nie, chrzan tam dalej. Tam dalej jest, ale już mało go jest. Nie, jego jak robisz, to bardzo łzy leje się. Ja już nie mogę. Wolę kupić w sklepie. Ale dawniej to tak, to dużo go używaliśmy*” [*No, chrzan is further out there [growing]. It is growing far away, but it is already disappearing. No, while you are making it, you are crying a lot. I cannot do it anymore. I prefer to buy it. But in the past, yes, it was used a lot*] (Lithuania, Polish woman, 74 years old).

**Fig. 9:**
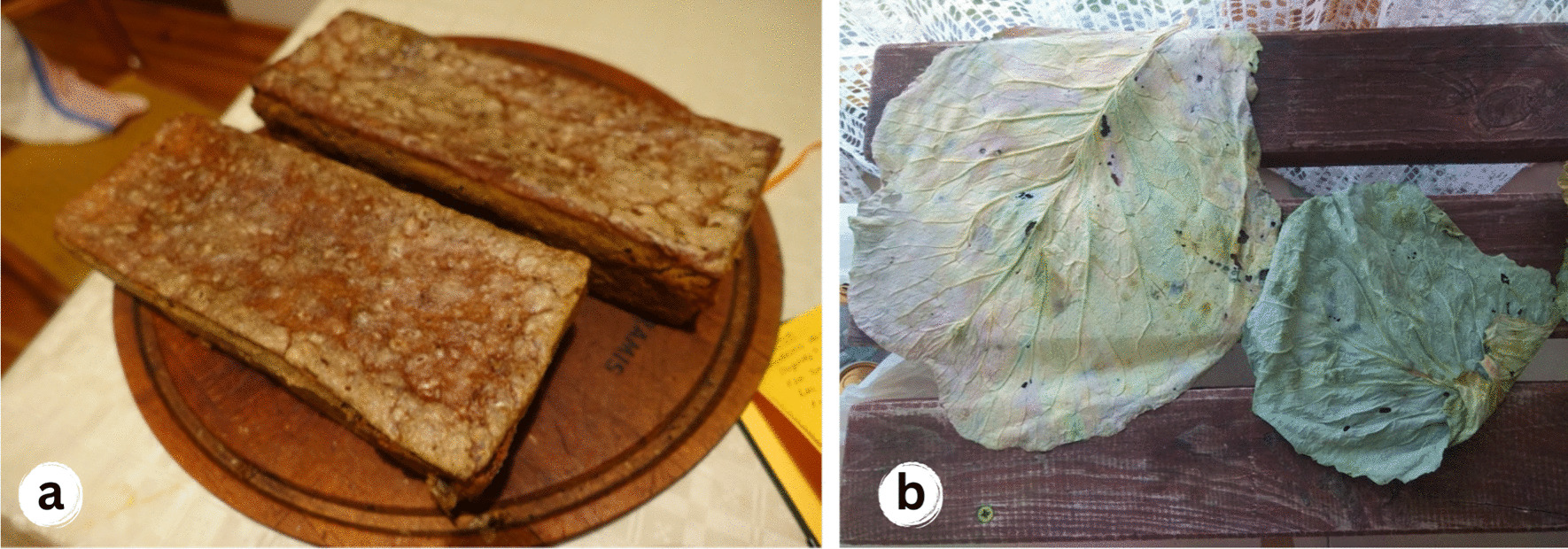
**a** Homemade bread baked on *Acorus calamus* leaves; **b** Drying of cabbage leaves to be used for baking bread and potato pancakes, Poland. Credits: Julia Prakofjewa, Poland, 2019

At the same time, the use of some taxa was attributed mainly to the past. For instance, the leaves of *Acorus calamus* and *Quercus robur* were mentioned in regional historical sources as being placed under bread while baking (see [37, 38]). Our fieldwork confirmed the recorded archival records. Interviewees from all three countries also mentioned the traditional use of fresh or dry leaves of *Acer platanoides and Brassica oleracea* for this purpose (Fig. 9). However, the tradition of making homemade bread has been gradually disappearing in the study area since the second half of the twentieth century. Nowadays, bread making is rarely mentioned in Poland and Lithuania and only in the context of choosing healthy food that is not bought from a store and with the possibility of using modern baking equipment.

*Stellaria media, Chenopodium album,* and *Heracleum sphondylium* were historically widely used for making sour and non-sour soups in the region (see [36, 37, 63]. However, among our interviewees, these taxa were primarily associated with famine foods from the war and post-war periods. Due to cultural stigmatisation [69] (Menendez-Baceta et al., 2012), nowadays, their use has gradually been abandoned: "*I heta (Stellaria media) taksama dobraja raślina. U dziacinstvie pamiataju źbirali, ale nie čuŭ, kab zaraz karystalisia. Heta paśla vajny bolš, jak galadali, baba raskazvała*” [*And it (Stellaria media) is also a good plant. I remember I collected it as a child, but I have not heard that people are using it now. It was used mainly after the war when people used to starve, my grandmother told me*] (Belarus, Polish man, 60 years old).

From an ecological perspective, if widespread cultivation ceases in some species, they will no longer naturalise in the wild, ultimately leading to the disappearance of their use in the long term. For example, *Cichorium intybus* and *Acorus calamus*, which rely on human intervention for their spread in a specific area, exhibit this pattern. Conversely, plant species whose expansion into nature is facilitated by cultivation have maintained their historical utilisation in the region over time, such as *Armoracia rusticana*, *Nepeta cataria*, and *Borago officinalis*.

*Knowledge decay* over time occurs when TEK is no longer valued or passed down to younger generations, leading finally to biocultural diversity loss [70]. In this flow variation, TEK is no longer practised. For example, *Vaccinium uliginosum* is one of the plants that has gone out of use within the studied region but remains vivid in the memory of our interviewees*.* Lithuanians and Poles attributed the discontinuation of this practice to the complete disappearance of this plant from the surrounding landscape. Although our interviewees have begun to forget its local name and details of its use, they still distinctly remember the taste and smell of the plant.

Nevertheless, many plants within this domain have historical evidence of use. For instance, *Calluna vulgaris* was documented as used as an additive to bread within the studied region in the 1930s (see[38]) and in Central Poland [63], which was confirmed by our fieldwork only in Lithuania.

We observed that knowledge decay was often related to the gender division of certain practices. For instance, collecting sap from trees is traditionally considered a male practice within the studied area, while collecting and preserving berries is more of a female practice. Consequently, knowledge can decay without a carrier of the practice: "*Vendzili miasa, sała, rybu. Da. Eta haspadar jak byŭ, mieŭ. I kapciŭ i kiłbasy i skvarku. A teraz jaho niama—to niama kamu. Spiecyjalnaja dreva, ale ja nie viedaju jakoje*” [*We smoked meat, lard, fish. Yes. It was my husband who had the smokehouse. He smoked sausages and lard. And now he is gone—there is no one to smoke. He put meat and smoked it with a special tree, but I do not know which*] (Belarus, Lithuanian woman, 82 years old).

The creation of new knowledge regarding the use of wild plants could be recognised as an *invention*. This may involve developing tools, techniques, or methods for managing natural resources, as well as the formation of new beliefs or attitudes about plants. We observed that the newly acquired knowledge of use and the intention to engage in culinary experimentation are closely connected. As people become more interested in exploring new flavours and ingredients, they are more inclined to seek out new plants to incorporate into their cooking practices. We found that both ethnic groups we studied in Poland and Lithuania exhibit a characteristic trait of having more intention to experiment with new plants.

**Fig. 10:**
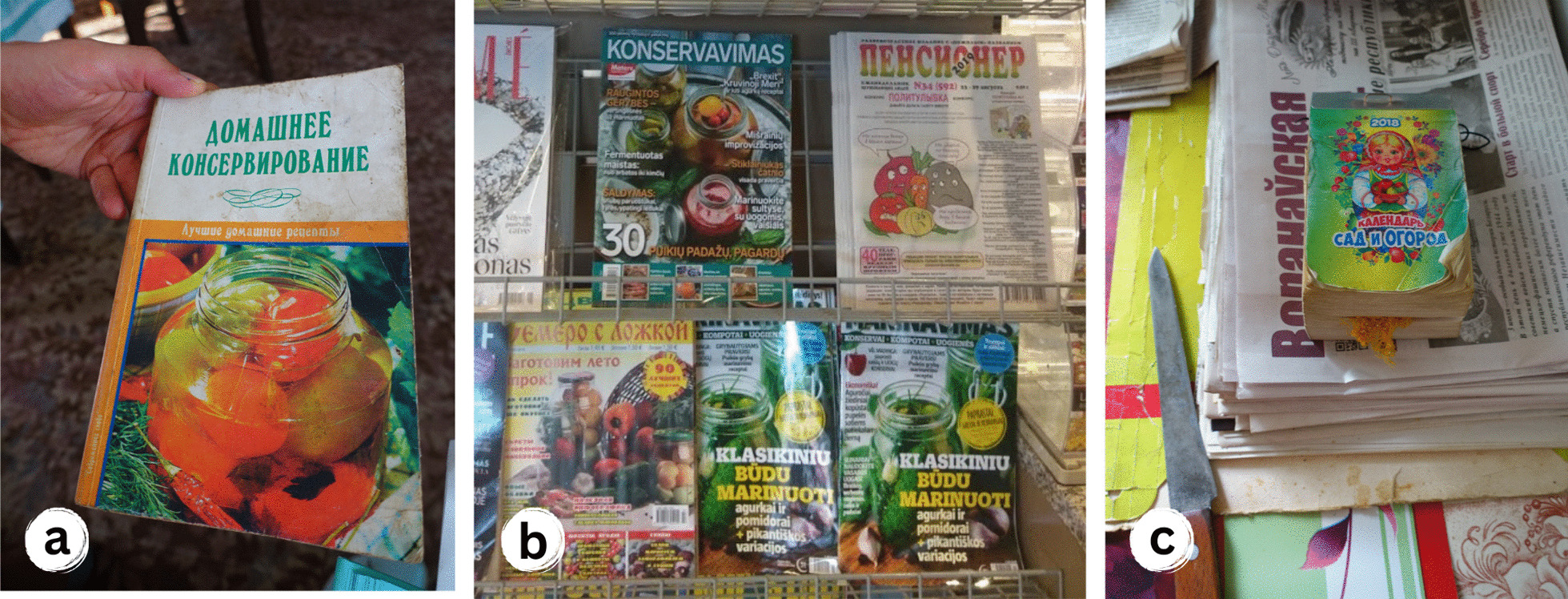
**a** Book about home conserving, Belarus; **b** Popular newspapers and magazines published in Lithuanian and Russian, introducing the new recipes for conserving and canning mentioned by our interviewees, for sale at a local news-stand, Lithuania; **c** Local newspapers and calendar as a popular source for new plant knowledge, Belarus. Credits: Julia Prakofjewa, 2018–2019

Our fieldwork showed that new uses originated mainly from popular literature and media in all three countries (Fig. 10). These sources remain essential to and trusted by older generations: *"Vo hladzi, heta maja knižačka. Tamaka ŭsio jest. Što tolki rabić—tamaka ŭsie jest. Jak sušyć, jak varyć, jak zamaražvać, jak kansierviravać, jak lačyć, jak što rabić. Tam usie jest"* [*Look, this is my book. There is everything in it. Everything you can do. How to dry, how to cook, how to freeze, how to conserve, how to cure, how to make. There is everything in this book*] (Belarus, Lithuanian woman, 78 years old). Among younger generations with more schooling (aged 40–65), a significant proportion of ethnobotanical knowledge is acquired through written and visual sources, in contrast to the mainly traditional and vertically transmitted knowledge of senior interviewees (aged 65–90). Older interviewees reported using plant-related books in Russian published during the Soviet Union era. The knowledge present in these books and previously widespread in Belarus and Lithuania primarily influenced specific areas such as novel preservation methods (e.g., the freezing of *Fragaria vesca*) and attributed medicinal properties to food plants (e.g., high vitamin content in *Urtica dioica* or *Rosa* sp.). According to our interviewees, the health benefits associated with *Cichorium intybus*, heavily promoted and advertised in the media in the 1990s, resulted in an increase in its use as a healthy coffee substitute in Poland and Lithuania: “*A to jest bardzo fajna roślina. Cykorium. Tak, kawę z cykorią robię. Tylko korzenia. Ale to ja przeczytałam gdzieś, że dobra jest. To (wiedza) nie od mamy, nie*” [*And it is a very nice plant. Chicory. Yes, I make coffee with chicory. Roots only. But I read somewhere that it is good. It (knowledge) is not from mom, no*] (Poland, Polish woman, 50 years old).

The process of renewing and restoring TEK that has been lost or forgotten over time may be identified as *knowledge revitalisation*. In the studied border region, an overarching trend we observed was a general shift towards a return to the use of wild plants for nutrition: "*No wiecie co. Konserwantów dużo teraz, ale też spróbujemy wrócić do tej przyrody. I ja, i młodzi ludzie. No to zależy, oczywiście. Najbardziej to młodzi*” [*You know what. We are using many preservatives now but also trying to return to nature. Both me and the young people. Well, it depends, of course. Mostly the young ones]* (Poland, Polish woman, 52 years old). One of the fundamental reasons why interviewees adopted new uses of wild food plants was their benefit to health. This trend is further reinforced by widespread media promotion of the "valuable properties" of wild plants: "*To jest aronia. Ja suszę na herbaty. Na wzmocnienia organizmu, bo to ona ma tam różne te. Ale bardzo smaczna jest. Przyniosę ci. Teraz przeczytałam o tym w książce. Aha, i w gazetach teraz to czytałam też*” [*This is aronia. I dry it for tea. Because it is used to strengthen the body. But it is delicious. I will bring you some. I read about it in a book. Oh, and I also read about that in the newspapers, too*] (Poland, Polish woman, 76 years old). Moreover, our research findings highlight that the preservation of plant usage is often observed in species that serve both medicinal and culinary purposes, as exemplified by *Tussilago farfara, Leonurus cardiaca*, and *Hypericum* spp.

**Fig. 11:**
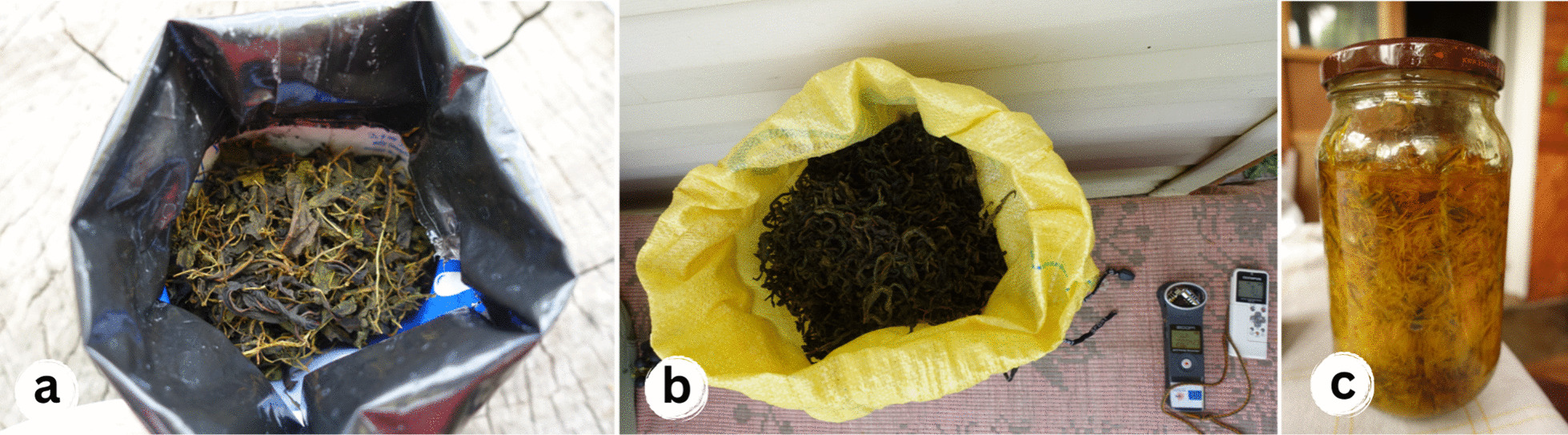
Recently acquired uses of wild plants for food in the studied region: **a** Fermented *Epilobium angustifolium* for recreational tea, Belarus; **b** Lithuania; **c** Syrup from the flowers of *Taraxacum officinale*, Poland. Credit: Julia Prakofjewa, 2018–2019

Highly promoted tea made from the fermented leaves of *Epilobium angustifolium* (Fig. 11 a-b) (see [23]) was tried and abandoned quickly within the entire studied region. This created the situation that the plant, without being broadly used historically, belongs simultaneously to recently acquired and past temporal dimensions and provides one excellent example of unsuccessful knowledge revitalisation in the region.

The active historical use of *Taraxacum officinale* in Belarus and Lithuania may be attributed to its promotion in books and women's magazines during Soviet times (see [56, 71]). However, a majority of our interviewees during the 1980s and 1990s attempted to make syrup ("dandelion honey") from the flowers of *Taraxacum officinale* (Fig. 11c) and now occasionally use the leaves in salads. This illustrates knowledge revitalisation, where innovation occurs at the level of plant parts used, as influenced by the literature. Despite its availability, *Taraxacum officinale* leaves did not gain popularity in Poland, possibly due to its culinary tradition of avoiding bitter flavours [72].

The movement of knowledge between continuous and recently acquired time domains could be identified as re-invention. This flow variation primarily occurs when ongoing knowledge and practices are adapted or modified to suit changing social, cultural, or economic contexts. For instance, if a traditional practice for preserving wild plants is no longer feasible due to advancements in technology or the availability of resources, re-inventing the practice can help keep the use of wild plants alive.

Specifically, in the study area, the use of the leaves of *Aegopodium podagraria* for sour and non-sour soups was recorded among Poles in historical sources (see [36, 37, 72]). We documented, in both studied communities, the use of these leaves for soup and salad proportionally more as a recently acquired practice and less as a continuous practice. The same trend was noted for *Borago officinalis*: “*O čia ogurečnik vadinasi. Čia kap neturi agurkų iš pavasaro, tai dedi saloton, tai būna ti kap su agurkais salota. Lapus. Nu [ji naudoja] Ale lietuviškai tai aš nežinau kaip jis vadinas*”. [*And this one is called ogurečnik. When you don’t have cucumbers in spring, you put it into the salad, and then the salad is like with cucumbers. Leaves [the part used]. Yes [she uses it]. But I do not know what it is called in Lithuanian*] (Poland, Lithuanian woman, 73 years old). Historically, the leaves of *Borago officinalis* were eaten with cream and salt (see [37]). However, we did not record anyone who has abandoned the use of this plant within their lifetime.

In the continuous time dimension, we observed specific plants mentioned by the interviewees as being utilised during their lifetime, leading us to identify this flow variation as *knowledge retention*. However, these taxa were not recorded in past uses of our sample. Nonetheless, we found historical evidence of their use within neighbouring territories. For example, historical records mentioned the use of *Tussilago farfara* as a food wrapping in some regions of southwestern Ukraine [36, 63, 72]. *Berberis vulgaris* was once reported as being consumed raw, cooked in soups, and used as a children's snack by Rostafinski's respondents [72]*,* but throughout Poland, the use of its fruits is common [63]. The continuous consumption of *Leonorus cardiaca* leaves as a recreational tea was likely related to their medicinal properties. However, the plants recorded here may have resulted from retrospective memory bias. Due to the way people's memories are biased [73], imprecisions, exclusions, or overstatements can occur in their recollections of previous events or occurrences. It is likely that the use of wild plants in this flow variation was limited to certain cultural traditions or ecological settings.

### ***The driving forces of changes in wild food plant use***

On the basis of the documented environmental discourse, it is reasonable to assume that various driving forces and proximate causes might have impacted the changes in the use of wild food plant knowledge within the studied border region over time. We found that numerous plant taxa remain stagnant, possibly indicating a conservative approach towards their utilisation for nutrition in the region. One of the primary factors possibly driving this pattern is the high resistance of LEK to change. This often results in a reluctance to embrace new technologies or methods that could facilitate more sustainable environmental management. If the knowledge currently available is sufficient to maintain life, there may not be much motivation for practitioners to create new knowledge or discover innovative applications: "*To jest (wiedza) wszystko od naszych dziadków*. *Jak one robili, tak i my robimy*" [*This is all (knowledge) from our grandparents. As they did, and so we are doing*] (Poland, Polish man, 48 years old).

On the other hand, one of the driving forces behind the suspension of the use of wild plants might be the absence of knowledge due to a lack of interest in this topic at a young age. For instance, one 72-year-old Polish woman from Lithuania shared her experience: "*Dawniej nie, no jak żyła jego matka, to zbierała roślinki, znała jakie co z tych. Ale ja nie interesowała się tym*" [*Not in the past, well, when his mother was alive, she collected plants, she knew for what [it was used]. But I was not interested in it*]. The majority of interviewees from Belarus and Lithuania emphasised that during their youth they had limited opportunities to learn as they were extensively engaged on collective farms (kolkhozes) and had little time for environmental educational pursuits.

During interviews, some of our senior interviewees struggled to understand our questions concerning the use of fruits, leaves, and especially roots of wild plants as seasonings: "*Kareńni ja nie panimaju takoha. Ja toža nie znaju etaha, jak što eci prypravy*" [*Roots*, *I don't really understand this. I also don't know what these seasonings are*] (Belarus, Polish woman, 73 years old). Most interviewees argued that their parents and grandparents did not use any seasonings in food, which they attributed to widespread poverty throughout the region, especially in the post-WWII period: "*A što rańše było, i da vajny i paśla asobienna, znajecie kak ludzi žyli biedna. Nie dumali pra prypravu. Eta sčaz pašła taja pryprava. Tahda nichto pra takoje nie znaŭ. I tak kak było—zažaryš i paješ, i dobra*" [*And what was before the war, and especially after it, you know how poorly people lived. No one thought about seasonings. Now seasoning is used. Then no one knew about it. And as it was—you cook and eat, and it is good*] (Belarus, Polish woman, 94 years old).

Knowledge flow may continue or cease depending on the availability of certain natural resources. Addressing the depletion of resources is one of the key factors attributed to using plants in studied countries with different environmental policies: "*Jezus, strasznie zmienił las się, był wielki piękny, powycinali, powycinali, powycinali. A teraz już takie co to teraz. Wo takie, wo takie. Co tam już teraz takiego już nie ma, bo co najlepszy las to wycięty*” [*Jesus, the forest has changed terribly. It was great and beautiful, but they cut it down, they cut it down, they cut it down. And now, what is it now? There are no such [food] plants there now because the best forest is cut down*] (Poland, Polish woman, 91 years old). Intense deforestation, land reclamation, the drainage of wetlands, and other environmental engineering projects have likely resulted in the disappearance of specific plant taxa within the studied region.

During the period between 1955 and 1995, which was characterised by intensive drainage activities in Lithuania and Belarus, significant and contentious actions were undertaken, destroying wetlands [74, 75]. Peat bog vegetation faced severe threats from land reclamation projects and the eutrophication of water bodies, influencing species composition negatively. More specifically, the decreased water levels have caused the proliferation of accompanying shrubs, compromising the vitality of *Vaccinium oxycoccos* and resulting in a significant decline in its populations [76]. Consequently, due to its extinction from the surrounding environment, the utilisation of this plant has largely been abandoned by our interviewees in Lithuania and Belarus: “*Na abłocie eta žuraviny. Chadzili ich, sabirali. Pravalivalisia, tam stanieš vo tak vo na etam vo—a tam vadzička pad spodam. Nu pravalivalisia ludzi. No sabirali. A ciapier užo nima tych bałotaŭ i tych jahad*” [*Cranberries grew in the swamps. We used to go and collect them. You could fall into the swamp because there is water under the surface. Well, people fell into the swamp but collected. And nowadays, there are no longer any swamps or berries*] (Belarus, Lithuanian woman, 74 years old).

When reflecting on the shifts in the utilisation of wild food plants, interviewees mainly highlighted changes in their values and attitudes towards such plants. These changes were particularly noticeable among older interviewees, as certain plants are no longer regarded with the same respect, leading to decreased motivation to collect and consume them. Additionally, alterations in dietary habits among younger generations were also mentioned as a contributing factor. However, the recent dying out of some uses (because they were not trendy anymore) was a popular explanation for the non-use of wild plants in Belarus.

Changes in food preservation methods and the industrialisation of food production during the study period have also impacted the utilisation of wild plants for food. The winter storage of wild plants has been gradually transforming since the introduction of modern technologies to the village in the latter half of the twentieth century. For instance, the introduction of freezers has simplified food preservation. At the same time, traditional techniques like smoking meat and fish and utilising wild plants for preservation are gradually decreasing across the entire region due to the widespread use of refrigerators and the decline of cattle farming.

Our observations revealed that the practice of jam-making is also decreasing in all three countries, as the younger generation perceives it as unhealthy because of its high sugar content. Nevertheless, preserving wild food plants for the winter and not using them later on was quite commonly recorded throughout the studied region. Interviewees indicated that throwing away unused jam made from wild berries is emotionally difficult, but they reluctantly do so. Also, they store new jars for the next season “*just in case*”. Some interviewees from Belarus and Lithuania were eager to offer us unused preserves. Thus, the practice of preserving plants is more vital than using them for food. This might be explained by traumatic famines in the past [77], which are still present in the minds of the people. The permanent food shortages that widely existed, despite the myth of Soviet food abundance [78], have resulted in culinary practices associated with the lack of food in territories occupied by the Soviet Union. 

Several of our interviewees have highlighted the fact that nowadays it is possible to buy everything in the shop, regardless of the season: "*Čaj prošče kupić, čym zavarvać. Viedajecie, eta treba nabrać, treba vysušyć. A to pryjšoŭ kupiŭ, i vypiŭ*" [*Tea is more accessible to buy than brew. You know, it has to be collected. Still needs to dry. And here you come and buy it and drink it*] (Belarus, Lithuanian woman, 82 years old). Thus, for them, there is no need to collect wild plants. At the same time, some emphasised the unsatisfactory quality of what can be bought, arguing for a return to foraging: *"A zašła ŭ mahazin kupić čaj, japonski niejki ci kitajski, i kak pačytała sastaŭ, i padumała, čaho pakupać, kitajski, jak u nas usie raście—treba viartacca"* [*And I went to the store to buy tea, some Japanese or Chinese, but as I read the composition, I thought—why buy Chinese tea, if everything grows here. We need to return (to nature)*] (Belarus, Polish woman, 62 years old).

While the re-invention of TEK can contribute to the preservation of biocultural diversity, it can also lead to controversy or conflict if it is perceived as inauthentic or disrespectful to the original traditional use. For instance, the use of the wood of cultivated *Malus* spp. for smoking meat was categorically perceived negatively by one of our interviewees from Belarus: "*Kapciłki byli raniej. Nu kapcili čem. Jałaviec. Takoje lasnoje znajecie. A ciapier jałaŭcom nie kapciać—ciapier ža jeść što-ta druhoje. Ci to jabłynia, ci etaj ci što. No. Eta ŭsie vydumki. Pravilna tolki jełaŭcom, niešta dzikaje—tady kaŭbaski ŭkuśnieńkija”* [*There were smokehouses before. Well, they smoked with something. Juniper. So, something from the wood, you know. And now they do not smoke with juniper—now it is something else. Or an apple tree from the garden or something else. But this is all fiction. The right one is only wild juniper, then the sausages are delicious*] (Belarus, Polish man, 86 years old). In some cases, the modified practice may lose its cultural significance or connection to the environment and thus become just a commercial product or tourist attraction.

Knowledge movement requires collaboration and the exchanging of ideas among local communities [79]. However, it may be hindered if there are barriers to collaboration, such as cultural or linguistic differences, political or institutional constraints, or a lack of communication channels [80]. The studied region has undergone some territorial changes associated with the establishment of borders, resulting in communication challenges for local communities and consequently changes in knowledge of the use of plants: “*Nie można, kochana, to była żelazna kurtyna. Ściana. Mówię, jak krowy przeszły do granicy, całe kolonie moich rodziców. Krowy urwie się, krowa przejdzie, gdzie chce, ale to było wojsko trzeba, meldować wojsku. Oni meldowali się na Litwę, stamtąd przychodzili wojskowi, dopiero można było przegonić te krowy i zabrać. To był problem. Przejścia nie było kiedyś tak. Gdzie tam nic, to była żelazna kurtyna, no”* [*You can't, dearest, it was the Iron Curtain. Wall. I told you, how our cows, my parents' whole herd, crossed the border. The cows broke out and went where they wanted. But we had to report this to our border guards. They reported it to Lithuania. And from there the military came and chased the cows back to our side. It was a problem. The border crossing was not like it is now. You could do nothing, it was the Iron Curtain*] (Poland, Polish woman, 72 years old). Interestingly, that the term "Iron Curtain" was used by the interviewee to emphasize the significant level of division between territories. Although the Iron Curtain typically refers to the borders between socialist and capitalist countries, in this context, it illustrates the perceived strict separation between the USSR and Poland.

### Mapping of the temporal dynamics of wild plant use

Our research findings align with prior ethnobotanical investigations that underscore the significance of considering LEK as a dynamic phenomenon [6, 81, 82]. Additionally, our study emphasises the importance of recognising the intricate temporal dimensions involved in knowledge circulation [83]. Through the analysis of a collection of environmental narratives, we closely documented the fluctuations in knowledge throughout our interviewees' lifetimes and observed the evolving patterns in their interactions with the natural environment. This examination enabled us to propose a conceptual model of knowledge circulation, facilitating a more profound comprehension of the temporal dynamics of wild plant use.

**Fig. 12:**
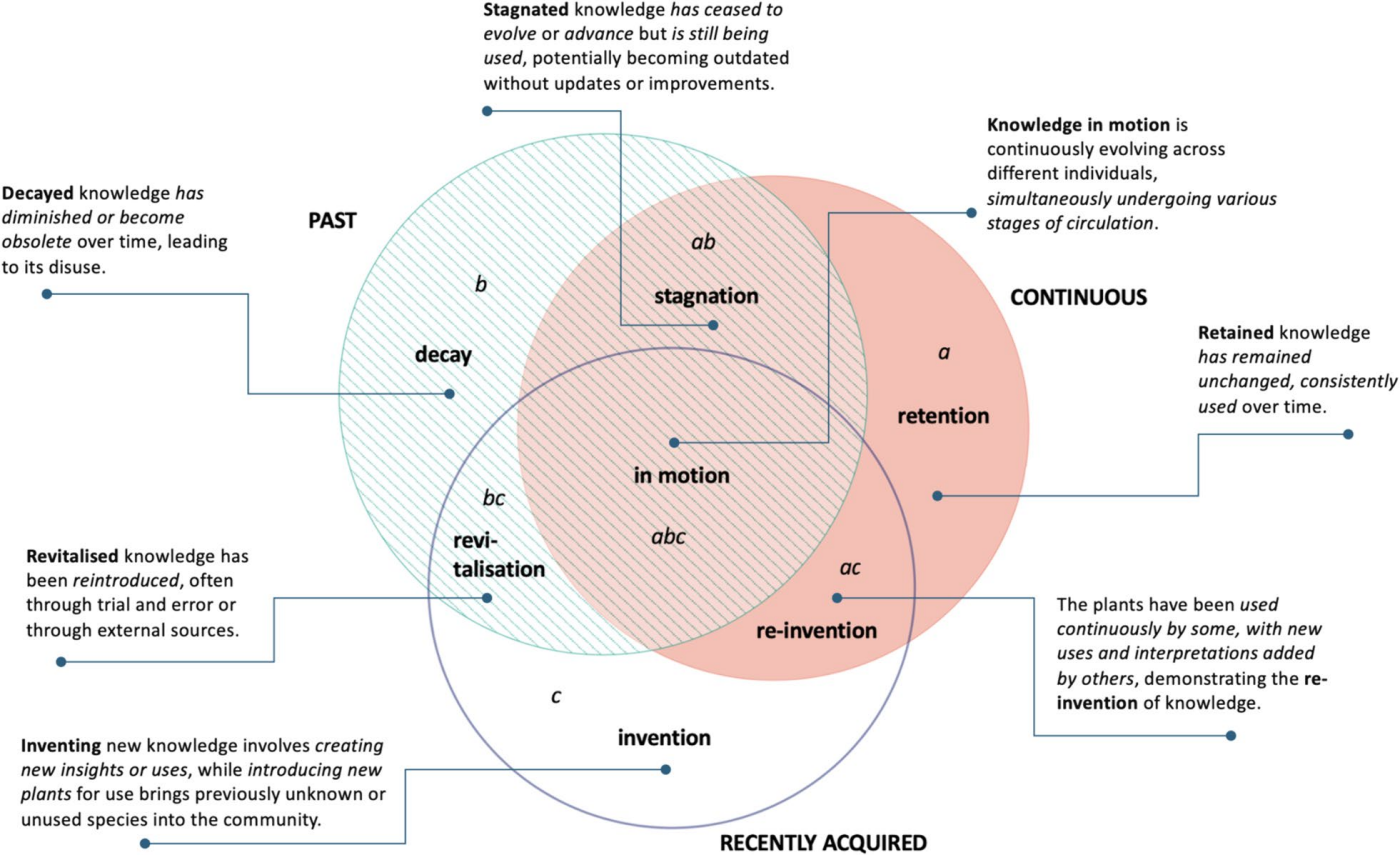
Conceptual model of plant knowledge circulation over time

Looking through a diachronic lens, we divided the documented knowledge into three significant temporal dimensions, representing past, continuous, and recently acquired uses. These dimensions were further classified into seven overlapping flow variations: retention, decay, invention, stagnation, revitalisation, re-invention, and knowledge in motion (Fig. 12).

Specifically, in the *knowledge in motion* (Fig. 12abc) flow variation, the use of wild plants circulates in all three temporal dimensions, which indicates that knowledge about plants has gone through various changes, including oblivion, being recently acquired, and continued use. It involves the active transmission of knowledge, contributing to continuity through innovation and retention despite decay. Hence, it refers to the process of the continuous sharing of uses among local communities.

Knowledge about taxa mentioned in both the continuity and past flow variations is stagnating (Fig. 12ab). For some interviewees, the use of wild plants continued, while for others, it had ceased. There is no place for new knowledge or experiments. In this flow variation, we see plants whose use is gradually eroding.

Knowledge that is not regularly used or applied among local communities is decaying (Fig. 12 b) over time. Here, the past uses of plants are still remembered but no longer practised, and so details on their use are lost or distorted. Such abandoned knowledge has little potential to be shared with the next generation.

The *retention* (Fig. 12a) flow variation refers to plants that are perceived as used uninterruptedly during the lifetime of interviewees. The retained knowledge is stable and conservative.

In the *re-invention* (Fig. 12ac) flow, some interviewees have been using the plants continuously and recently adopted by others. This flow variation is the most open to changes and innovations.

The *invention of knowledge *(Fig. 12c) is an ongoing process of introducing new plants or uses among local communities. It entails discoveries through trial and error and is often based on media promotion. These inventions are not related to continuous uses or based on TEK.

The *revitalisation of knowledge* (Fig. 12bc) occurs when once abandoned plants are reintroduced through additional sources of information, innovation, or new application. For some interviewees, the use of those plants has stopped, while others recognised them as new.

## Conclusions

By studying the temporal dynamics of wild food plant use in the Polish-Lithuanian-Belarusian borderland, we have gained valuable insights into how diverse local communities interact with their surrounding environment over time. The changes were driven by various factors, including shifts in environmental conditions, cultural practices, economic considerations, and changes in personal values and attitudes. This research introduces a novel model for tracking and naming changes in relationships between people, knowledge, and nature.

The results serve as a foundation for the development of community-oriented tools for environmental education and the promotion of sustainable conservation strategies. Acknowledging that LEK moves in multiple directions can be beneficial for fostering biocultural diversity. Specifically, it can lead to increased innovation and creativity in the use of plants. Unhindered knowledge circulation can also help preserve TEK that may be at risk of being lost. Furthermore, transmitting knowledge enables local communities to learn from each other's experiences and adopt the most suitable practices from various fields or cultures, particularly in cross-border contexts. It is essential to highlight that losing knowledge does not necessarily mean it is gone forever. Sometimes, efforts can be made to recover lost knowledge through research, documentation, and collaboration with local communities that still possess that knowledge.

## Data Availability

All data are available in this publication.

## Abbreviations

LEK: Local ecological knowledge
TEK: Traditional ecological knowledge
SE: South-eastern
NW: North-western
NE: North-eastern
DUR: Detailed use report(s)
PL: Poland
LT: Lithuania
BY: Belarus
WWII: World War II
USSR: The Union of Soviet Socialist Republics
